# Recent Advances in Fungal Infections: From Lung Ecology to Therapeutic Strategies With a Focus on *Aspergillus spp.*

**DOI:** 10.3389/fmed.2022.832510

**Published:** 2022-03-21

**Authors:** Fabio Palmieri, Angela Koutsokera, Eric Bernasconi, Pilar Junier, Christophe von Garnier, Niki Ubags

**Affiliations:** ^1^Laboratory of Microbiology, Institute of Biology, University of Neuchâtel, Neuchâtel, Switzerland; ^2^Faculty of Biology and Medicine, University of Lausanne, Service de Pneumologie, Centre Hospitalier Universitaire Vaudois (CHUV), Lausanne, Switzerland

**Keywords:** chronic respiratory disease, microbiome, mycobiome, aspergillosis, live biotherapeutic products, disease management, environmental interference

## Abstract

Fungal infections are estimated to be the main cause of death for more than 1.5 million people worldwide annually. However, fungal pathogenicity has been largely neglected. This is notably the case for pulmonary fungal infections, which are difficult to diagnose and to treat. We are currently facing a global emergence of antifungal resistance, which decreases the chances of survival for affected patients. New therapeutic approaches are therefore needed to face these life-threatening fungal infections. In this review, we will provide a general overview on respiratory fungal infections, with a focus on fungi of the genus *Aspergillus*. Next, the immunological and microbiological mechanisms of fungal pathogenesis will be discussed. The role of the respiratory mycobiota and its interactions with the bacterial microbiota on lung fungal infections will be presented from an ecological perspective. Finally, we will focus on existing and future innovative approaches for the treatment of respiratory fungal infections.

## Respiratory Fungal Infections

Fungal pathogens are estimated to lead to more than 1.5 million deaths every year worldwide, with a global burden exceeding one billion (1). Despite this, the issue of fungal pathogenicity has been largely neglected (2, 3). Over the past two decades, the prevalence of invasive fungal diseases has increased considerably (1). This has also been acknowledged in the case of healthcare-associated invasive fungal infection (4, 5), for which a call to action was recently issued by the scientific community (6). Moreover, the increased prevalence of invasive fungal diseases correlates with an increasing number of vulnerable at-risk patients, which include among others, immunosuppressed individuals due to transplants, AIDS, cancer, corticosteroid therapies or autoimmune diseases, or patients undergoing major surgery (1, 7).

The most prevalent human fungal pathogens are the airborne opportunists *Aspergillus* spp., *Cryptococcus* spp., and *Pneumocystis* spp., as well as the human-associated commensal and polymorphic fungal species *Candida albicans* (7, 8). These fungi are responsible for more than 90% of all reported fungal disease-related deaths (9). The latest estimates of the annual burden of fungal diseases amount to more than 14 million cases for all diseases within the pulmonary aspergillosis spectrum, over 200,000 cases of cryptococcal meningitis, 500,000 cases of *Pneumocystis jirovecii* pneumonia, 700,000 cases of invasive candidiasis, and over 10 million cases of asthma with fungal sensitisation (1, 9, 10).

In the current review, we will first look at the full spectrum of diseases caused by fungi of the genus *Aspergillus*, with a particular focus on the pathogenesis and the underlying immunological mechanisms. Then, lung ecology, and more specifically the interaction of the respiratory mycobiota (fungal composition) with the bacterial microbiota and the virome will be discussed in the context of fungal infection. Finally, we will discuss the current therapeutic approaches, as well as future perspectives in therapeutic strategies for the fight against pulmonary aspergillosis.

### Pulmonary Aspergillosis: A Wide Spectrum of Diseases

*Aspergillus* spp. are the most frequently isolated filamentous fungi in humans and animals (11–13). These primarily saprotrophic fungi are widespread in the environment and can be found in soil and decaying biomass, especially in compost piles, where they participate in the degradation of organic matter (14–16). Their wide environmental distribution can be explained by the competitiveness and adaptability of the *Aspergillus* genus (17). Indeed, *Aspergillus* spp. are able to use multiple organic substrates and adapt to a broad range of environmental conditions (12). These fungi show a remarkable phenotypic plasticity in their ecology and stress-responses, which are believed to be at the basis of the success of *Aspergillus* spp. as opportunistic pathogens.

**FIGURE 1:**
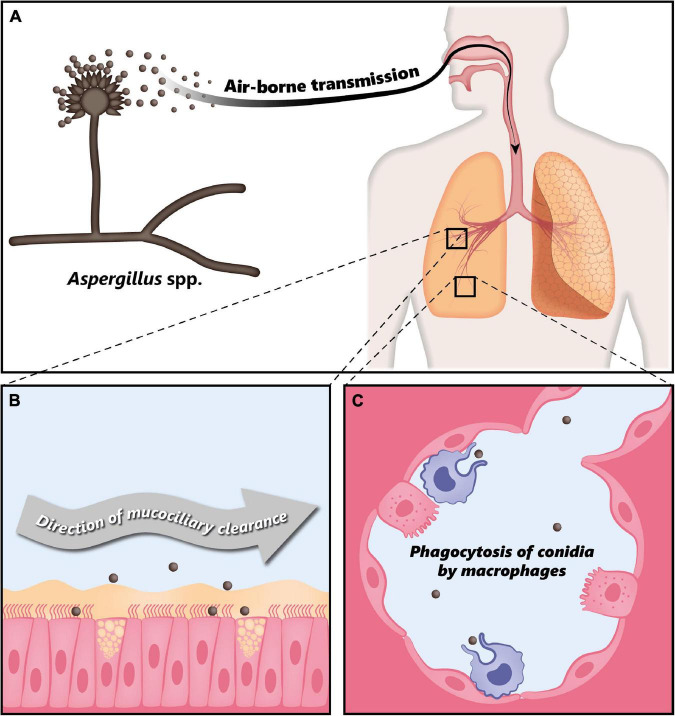
Clearance of *Aspergillus* conidia in the immunocompetent host. **(A)** *Aspergillus* spp. conidia are transmitted through air and every individual inhales thousands of conidia every day. **(B)** In the immunocompetent host, most of the inhaled conidia are trapped by the mucus layer secreted by the tracheal and bronchial epithelium, and are efficiently eliminated through mucociliary clearance. **(C)** Due to their small size, conidia can eventually reach the alveoli, where they are phagocytosed by alveolar macrophages. Modified from Palmieri (170).

Fungi of the genus *Aspergillus* are associated with a large variety of clinical manifestations ranging from allergic reactions to life-threatening invasive infections. Such infections are generally caused by *Aspergillus fumigatus*, *Aspergillus flavus*, *Aspergillus niger*, *Aspergillus nidulans*, and *Aspergillus terreus*, with *Aspergillus fumigatus* being responsible for 90% of the reported cases (12). Respiratory infections due to *Aspergillus* spp. are caused by inhalation of airborne conidia, i.e., asexual spores (14). These fungi produce large quantities of small airborne conidia with a size of 2–5 μm in diameter (15, 18), whose concentration can range from 1 to 100 per m^3^ in air, but can reach up to 10^8^ per m^3^ in some environments (13). A human inhales approximately 100–1,000 conidia per day, which can reach the lung alveoli due to their small size (15). In immunocompetent individuals, inhaled conidia are usually efficiently cleared either by mucociliary movement or through phagocytosis by macrophages (Figure 1) (18). However, depending on the immunological status of the host, *Aspergillus* spp. can lead to a variety of pathologies (12).

**FIGURE 2:**
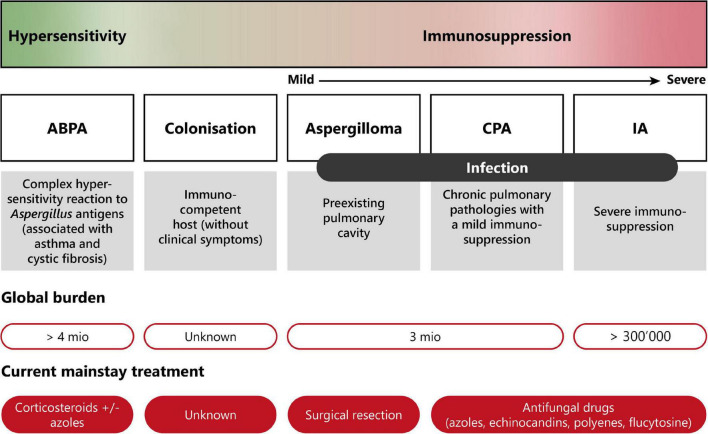
Disease spectrum of pulmonary aspergillosis. This diagram summarizes the diverse pathologies caused by *Aspergillus* spp., due to improper elimination of conidia by mucociliary clearance or macrophage phagocytosis following inhalation. Depending on the immune status of the host, pulmonary aspergillosis can range from allergic reaction (hypersensitivity) to life-threatening invasive infection (severe immunosuppression). Moreover, current burden and treatment information are indicated. ABPA, allergic bronchopulmonary aspergillosis; CPA, chronic pulmonary aspergillosis; IA, invasive aspergillosis. Modified from Palmieri (170).

Pulmonary aspergillosis is classified into three different groups with distinct clinical manifestations (11, 13, 19). The disease spectrum of pulmonary aspergillosis spans from hypersensitivity responses (asthma or allergic bronchopulmonary aspergillosis—ABPA), to colonization (i.e., presence of the fungus without any clinical, radiological or laboratory indications of active fungal disease), to infection (chronic or invasive aspergillosis). Figure 2 presents a diagram showing the disease spectrum of pulmonary aspergillosis depending on the host status.

#### Hypersensitivity Responses

Although other fungi can cause allergic bronchopulmonary mycoses (ABPM), the vast majority of hypersensitivity responses are associated to *Aspergillus* spp. These can range from fungal asthma to allergic bronchopulmonary aspergillosis (ABPA), the latter being a complex type I, III, and IV hypersensitivity response observed notably in patients with cystic fibrosis (CF) or chronic asthma (13). Hypersensitivity to *Aspergillus* is characterized by high levels of *Aspergillus*-specific IgE (19). ABPA affects close to 5 million patients worldwide (1, 10).

#### Colonization

In immunocompetent hosts, *Aspergillus* spp. may colonize the lungs without any clinical manifestations (10). In a study from Soubani and colleagues where *Aspergillus* spp. were isolated from sputum samples of 66 elderly hospitalized patients, 92% cases were determined to be *Aspergillus* colonization and only 4.5% fulfilled the criteria of invasive aspergillosis (20). Appropriate diagnostics and close monitoring should be considered in order to discriminate simple colonization from invasive infection (10, 11, 19). In immunocompromised patients, prior fungal colonization in the lower respiratory airways is considered an important risk factor for the development of invasive aspergillosis (10).

#### Chronic Pulmonary Aspergillosis

*Aspergillus* spp. can also cause a chronic, non-invasive form of infection called chronic pulmonary aspergillosis (CPA). One form of CPA is aspergilloma, which is characterized by the proliferation of the fungus inside a pre-existing cavity, leading to the development of a fungus ball (21). Aspergilloma typically occurs in immunocompromised patients previously suffering from lung pathologies such as tuberculosis, lung abscess, cysts, or tumors (21). Another form is chronic cavitary pulmonary aspergillosis, also called chronic necrotizing aspergillosis or complex aspergilloma. This is an inflammatory form of the infection characterized by the production of serum IgG antibodies directed to *Aspergillus*, elevated acute-phase inflammation markers, and the absence of pulmonary or vascular invasion. CPA usually occurs in immunocompetent or mildly immunosuppressed patients. CPA, including aspergilloma, is estimated to affect more than 3 million people worldwide (1).

#### Invasive Pulmonary Aspergillosis

On the other side of the disease spectrum, invasive pulmonary aspergillosis (IPA) is the most severe and life-threatening form of *Aspergillus* infection occurring in immunosuppressed patients. IPA affects more than 300,000 patients annually and its mortality rate ranges from 30 to 80% (1, 10). IPA is characterized by the invasion of the lung tissue by *Aspergillus* hyphae, which can be followed by angioinvasion and dissemination to other organs in patients with prolonged neutropenia (22). Other at-risk patients include individuals who underwent hematopoietic stem cell transplantation, solid-organ transplantation, prolonged corticosteroid therapy, or those who have AIDS (11, 13, 19). *Aspergillus* spp. are the most common opportunistic fungal pathogens causing invasive pulmonary aspergillosis in lung transplant recipients, with an incidence of 40.5 cases per 1,000 patients annually, despite the use of prophylactic antifungal treatments (23). Invasive aspergillosis most commonly occurs within 1 year after transplantation, with the majority of the cases reported within the first 6 months (23, 24).

### Fungal Infections in Chronic Respiratory Diseases

In healthy individuals, innate immune responses and specifically macrophages and ciliated bronchial epithelial cells contribute to the efficient clearance of fungal conidia. In patients with chronic respiratory diseases, such as chronic obstructive pulmonary disease (COPD), asthma, and CF, these clearance mechanisms are impaired predisposing to fungal colonization and infection. In the following paragraphs, we will summarize the role of fungal pathogens in specific pulmonary diseases, focusing especially on *Aspergillus* spp.

#### Chronic Obstructive Pulmonary Disease

Chronic obstructive pulmonary disease patients are often affected by exacerbations due to bacterial infections. The use of long-term inhaled corticosteroids and courses of oral steroids to treat exacerbations can predispose to fungal colonization and infection, as these treatments lead to impaired host immunity (25–27). In addition, in COPD, environmental fungal sensitization has been associated with frequent exacerbations (28). Bafadhel and colleagues reported that positive cultures for filamentous fungi are common in COPD, however, this finding was not related to exacerbations (26). Moreover, *A*. *fumigatus* sensitization was associated with poor lung function and, interestingly, patients with a positive *A. fumigatus* culture were on higher inhaled corticosteroid doses and had higher total and percentage sputum neutrophil counts (26). Recently, Tiew and colleagues evaluated the airway mycobiome in COPD patients in a multicenter study and observed that COPD patients with very frequent exacerbations (≥3 per year) had an increased number of fungal interactions (29), which is suggestive of a more complex mycobiome. Using unsupervised hierarchical clustering of the COPD mycobiome, the authors reported two distinct patient clusters with variable clinical outcomes: the first cluster was characterized by *Saccharomyces* and increased symptoms, whereas the second cluster was characterized by *Aspergillus*, *Curvularia*, and *Penicillium* and demonstrated poorer clinical outcomes with increased exacerbations and higher mortality (29).

Innate immune cells and macrophages in particular play an important role in the first line of pulmonary host defense. In COPD patients, it has been demonstrated that alveolar macrophages exhibit reduced phagocytic capacity (30, 31). Monocyte-derived macrophages from both smokers and COPD patients were shown to be defective in their phagocytic and pro-inflammatory cytokine responses following *A. fumigatus* exposure (32). This impairment in macrophage function may consequently contribute to fungal germination, dissemination and infection, and lung damage in COPD patients. There is an increasing interest in understanding the direct influence of fungal colonization and infection on COPD pathogenesis and exacerbations. However, future research should continue to consider the indirect effects of bacterial composition alterations on fungal community composition in the lung through inter-kingdom interactions, and the potential consequences for COPD patients.

All diseases within the aspergillosis spectrum can be found in COPD patients although their prevalence differ. Tiew and colleagues have recently reviewed this topic in depth (33).

#### Asthma

Genetic and environmental factors drive asthma development, progression and risk for exacerbation. Changes in fungal community composition in the gut are associated with susceptibility to develop asthma in humans (34, 35), however, causality has not been established yet. Analysis of the airway mycobiome in asthma patients (fungal-sensitized and non-fungal sensitized) and healthy controls indicated that both the sputum and bronchoalveolar lavage (BAL) mycobiome was dominated by three species: *A. fumigatus, C. albicans*, and *Mycosphaerella tassiana*, irrespective of health status (36). Interestingly, other fungi such as *Aspergillus tubingensis*, a member of the *A. niger* species complex, was also prominent in the BAL fluid. Alterations in the balance of fungi detected in the lung were found to be associated with several disease markers, including asthma status and duration, and inflammatory biomarkers.

Fungi can play an important role in asthma development as fungal colonization and sensitization often take place in early life. Moreover, fungi are predominant triggers of asthma exacerbations. Environmental presence of fungi, such as *A. alternata*, in house dust has been associated with active asthma symptoms (37). In addition, a meta-analysis of seven studies revealed that indoor presence of *Cladosporium*, *Alternaria*, *Aspergillus*, and *Penicillium* was associated with enhanced asthma exacerbations in both children and adults (38). Descriptive and mechanistic studies have started to reveal the influence of alterations in the gut bacterial and fungal composition on asthma development (discussed in section “Gut-Lung Axis”). However, the influence of a change in the composition of the pulmonary bacterial community due to asthma, on the susceptibility to develop a respiratory fungal infection, and subsequent asthma exacerbation, is currently unexplored.

#### Cystic Fibrosis

Cystic fibrosis is a rare autosomal recessive disorder that causes severe damage to the lungs, digestive system and other organs. The microenvironment in the lungs of CF patients, which is characterized by depletion of the airway surface liquid layer leading to impaired mucociliary clearance, is ideal for microbial colonization (39). Bacterial pathogens, and most commonly *Pseudomonas aeruginosa*, are known contributors to disease progression and exacerbations (40). Although fungi are often isolated from the lower airways of CF patients, the clinical impact of their presence and especially for the development of non-allergic fungal disease is poorly understood (41, 42). The most frequently detected fungi in the airways of CF patients are *Aspergillus* spp., notably *A. fumigatus*, and *Candida* spp. (43). The prevalence of *Aspergillus* colonization was highest in adolescents and young adults (44). Risk factors for *Aspergillus* colonization in people with CF include age and the use of inhaled corticosteroids and antibiotics (44). Moreover, aggressive use of antibiotics have been suggested to contribute to the increase in fungal colonization (45). This suggests that inter-kingdom interactions may be important in containing fungal colonization in the respiratory tract.

Interestingly, *A. fumigatus* colonization in people with CF is often preceded by *P. aeruginosa* infection (46). In patients infected with both *A. fumigatus* and *P. aeruginosa*, more severe clinical outcomes have been observed when compared to those infected with *P aeruginosa* alone (47). *P. aeruginosa* can have both antifungal and growth stimulating effects on *A. fumigatus* resulting in: (1) inhibition, (2) reciprocal antagonism, and (3) cooperation (48), which we will briefly describe here.

The main mechanisms by which *P. aeruginosa* can inhibit *A. fumigatus’* growth is via the release of phenazines, including pyocyanin, phenazine-1-carboxamide, 1-hydroxyphenazine, and phenazine-1-carboxylic acid. Phenazines are small diffusible quorum-sensing molecules, which easily penetrate *A. fumigatus* conidia, and are considered a strong virulence factor of *P. aeruginosa* (49–51). The quorum-sensing system allows bacteria to assess cell density and to regulate physiological activities accordingly, which consequently modulate the pathogenicity of the microorganisms (48, 52). Moreover, nutrient availability and competition is also involved in this inhibitory process. As an example, *P. aeruginosa* and *A. fumigatus* can both compete for the utilization of iron as a central nutrient for their survival (53). Although *P. aeruginosa* has a variety of fungicidal mechanism, alterations in these fungicidal capacities have been observed in clinical isolates from CF patients (52, 54), indicating that there can be shifts between inhibition and cooperation. Several cooperative interactions have been described which stimulate growth and potentially contribute to disease progression. Phenazines can stimulate fungal growth via increasing iron bioavailability (50). Moreover, pyochelin, a siderophore, can be used by *A. fumigatus* as a ferrochelator. Interestingly, dimethyl sulfide, a volatile organic compound released by *P. aeruginosa*, can communicate with *A. fumigatus* and create a positive growth environment resulting in stimulation of fungal growth (48, 55).

The Climax-Attack model (CAM) is a theory which has been proposed for CF a few years ago grounded on basic ecological principles (56). In this theory it is postulated that there are two major functional communities in CF pulmonary disease. The attack community consists of transient viral and microbial populations that induce strong innate immune responses. Consequent alterations in the immune response create a microenvironment that facilitates a climax chronic community having a reduced growth rate and being inherently resistant to antibiotic therapy (56, 57). Soret and colleagues provided important information on the involvement of the mycobiome in the CAM model in CF pulmonary exacerbations. They inferred an inter-kingdom network by plotting bacterial genera significantly correlated with at least one fungal genus and vice versa. Network analysis revealed three main clusters organized around *Aspergillus*, *Candida* and *Scedosporium* genera (57). The positively correlated OTUs predicted interactions of these three fungal genera with bacteria belonging to *Capnocytophaga*, *Parvimonas*, *Streptococcus*, or *Veillonella*. In addition, these interactions were assessed using *in vitro* co-cultures between *A. fumigatus* and *Streptococcus mitis* or *Streptococcus oralis* and confirmed that both *S. mitis* and *S. oralis* enhanced *A. fumigatus* growth. Such translational studies in which principles from ecology are used to understand disease can potentially form the basis for the future development of therapeutic strategies to combat exacerbations.

### SARS-CoV-2 Co-infection

The current SARS-CoV-2 coronavirus pandemic created the perfect arena for the establishment of opportunistic fungal co-infections. The use of high dose systemic glucocorticoids, which are widely used as an anti-inflammatory medication for COVID-19 (58–60), together with epithelial cell damage in the lung following SARS-CoV-2 infection, expose patients to opportunistic fungal infections, such as COVID-19 associated invasive pulmonary aspergillosis (CAPA) (61). To date, over 100 cases of CAPA have been reported (61). Moreover, invasive mucormycosis, also known as “black fungus,” has been largely reported in convalescent COVID-19, particularly in India and other Asian countries, in patients with uncontrolled diabetes mellitus or immunosuppression (62). The main reason for the increase in invasive mucormycosis cases has been pointed out to be the elevated iron levels in the serum of convalescent COVID-19 patients (62). Complementary to dexamethasone, Tocilizumab, an IL-6 receptor antagonist, has been widely used to treat COVID-19 in critically ill patients (63–65). IL-6 plays a critical role not only in the cytokine storm in severe COVID-19 (63, 65), but also in the innate immunity against fungal pathogens such as *A. fumigatus* (66). Accordingly, IL-6 inhibition has been associated with an increase in secondary infections in COVID-19 patients (67). A recent case report described an invasive *Aspergillus* infection in a COVID-19 patient following treatment with tocilizumab (65). Moreover, IL-6 inhibiting drugs may predispose COVID-19 patients to invasive mucormycosis (62).

## Mechanisms of Fungal Infections: From Innate Immune Response to pH Modulation

### Innate Immunity Against *Aspergillus* spp.

Despite constant exposure to *A. fumigatus* conidia, most people do not develop fungal disease. This suggests an efficient clearance of the conidia by the innate immune system in immunocompetent individuals before the adaptive immune system is activated (18). Upon inhalation, most resting conidia arriving in the respiratory tract are deposited against the airway fluid, due to turbulent airflow caused by the branching pattern of the respiratory tract (18). The trapped conidia are then removed by the ciliary action of the respiratory epithelium, which is the first line of defense in the lung (18, 68).

#### Host Recognition of Fungal Pathogen-Associated Molecular Patterns

Due to their small size, some of the inhaled conidia can reach the respiratory alveoli. After 4–5 h, resting conidia become swollen, and if not cleared, germinate and form hyphae within 12–15 h after arrival into the lungs (18). The maturation of conidia triggers a morphological change leading to the loss of the thin hydrophobic RodA protein layer, thus exposing the immunogenic components of the inner cell wall (69–71). These cell wall pathogen-associated molecular patterns (PAMPs) include polysaccharides such as β-D-glucan, mannan, chitin, and galactomannan, all of which are recognized by different pattern recognition receptors (PRRs) (15, 72). *A. fumig*atus conidia and hyphae can be recognized by the host via both soluble (pentraxins, complement proteins, and pulmonary collectins) and cell-associated microbial PRRs [Toll-like receptors (TLRs) and C-type Lectin receptors (CLRs)] (73, 74).

Pentraxins, such as pentraxin-3 (PTX3), are secreted by various cells, including neutrophils, dendritic cells, mononuclear phagocytes, and pulmonary epithelial cells (75). They bind to galactomannan on *A. fumig*atus conidia and facilitate recognition by phagocytes such as alveolar macrophages (76, 77). In addition, pulmonary collectins include lung surfactant proteins A and D and serve as opsonins. They bind to *A. fumig*atus conidial carbohydrate structures in a calcium-dependent manner. Surfactant proteins A and D have also been shown to promote the agglutination of conidia and their binding to neutrophils and alveolar macrophages, and improve the phagocytosis and killing of conidia by neutrophils (78).

Toll-like receptors recognition of pathogens triggers a signaling cascade leading to the activation of transcriptional factors such as NF-κB, which controls the expression of pro- and anti-inflammatory cytokines and chemokines (79). The universal adaptor molecule MyD88 has been shown to play a significant role in the signaling of TLRs, which induce the production of various inflammatory cytokines and reactive oxygen species (79). TLR2 and TLR4 have been implicated in the recognition of *A. fumig*atus conidia and hyphae (80). However, available data concerning their roles in *A. fumig*atus-associated immunity are conflicting. Indeed, the *A. fumigatus*-associated PAMPs for TLR2 and TLR4 remain undetermined. TLR9 has also been shown to play a role in innate immunity against *A. fumigatus* by recognizing fungal unmethylated CpG DNA (81). Dectin-1 is a CLR primary receptor that recognizes fungal β-glucan and that is essential for the mediation of the proinflammatory response (80), and is widely expressed on innate immune cells including macrophages, dendritic cells, and neutrophils (82–84). Dectin-1 can also induce the expression of the anti-inflammatory cytokine IL-10, indicating its dual role in modulating the inflammatory response (85). Dectin-2, another type of CLR, has recently been shown to be implicated in the innate immune response against *A. fumig*atus. Macrophages and dendritic cells express Dectin-2 and recognizes α-mannan in the fungal cell wall’s outer layer. Accordingly, in response to *A. fumigatus* infection, alveolar macrophages upregulate Dectin-2. Moreover, Dectin-2 was shown to mediate an NF-κB-dependent proinflammatory response against swollen conidia (80). Finally, DC-SIGN is expressed at the surface of dendritic cells and some macrophages, and binds to *Aspergillus* conidia via the recognition of fungal galactomannan (18). Genetic polymorphism in the above-mentioned PRRs, as well as in cytokines, chemokines, and immune receptors genes, has been associated with an increased susceptibility to pulmonary aspergillosis (86–88). For instance, the Dectin-1 variant Y238X has been shown to impair the production of several cytokines such as IFN-γ and IL-10 by human peripheral mononuclear cells, leading to an increased susceptibility to invasive aspergillosis in patients receiving hematopoietic stem cell transplantation (HSCT) (89). Furthermore, the Asp299Gly polymorphism in TLR-4 is highly associated with chronic cavitary pulmonary aspergillosis (86).

#### Cellular Immune Responses

##### Clearance of *Aspergillus* Conidia by Innate Immune Cells and Epithelial Cells

Alveolar macrophages (AMs), neutrophils and epithelial cells constitute the first line of defense against inhaled *A. fumigatus* conidia (18). Alveolar macrophages phagocytose and kill conidia either via oxidative mechanisms through the generation of reactive oxygen species (ROS), or by non-oxidative mechanisms through phagosomal acidification (80). Corticosteroids have been shown to impair the capacity of AMs to kill conidia (18). Neutrophils were initially thought to kill hyphae exclusively, however, they have also been essential in killing germinating conidia. Neutrophils bind and phagocytose swollen conidia to trigger respiratory burst and degranulation. While the size of the hyphae prevents phagocytosis, direct contact with neutrophils can induce oxidative and non-oxidative mechanisms to damage the hyphae (18). Moreover, respiratory epithelial cells, i.e., bronchial and alveolar epithelial cells, as well as endothelial cells, have been shown to participate actively in the innate immune response against *Aspergillus fumigatus* strains Af293 and CEA10 by phagocytosing and killing conidia *in vitro* (18, 90–94). Furthermore, human peripheral blood monocytes have also been shown to internalize *Aspergillus* conidia and inhibit their germination and hyphal growth (95). Interestingly, both classical (CD14^+^CD16^–^) and non-classical monocytes (CD14^+^CD16^+^) were found to successfully internalize conidia (96). However, only classical monocytes were able to inhibit *Aspergillus* hyphal development (96). Lastly, dendritic cells (DCs) also have well-documented roles in the defense against *A. fumigatus*. Immature DCs (iDCs) have been shown to phagocytose opsonized and non-opsonized conidia and hyphae, both of which are recognized through PRRs such as Dectin-1, among others. TNF-α, IL-6, IL-12, IL-1α, and IL-1β are the central proinflammatory cytokines produced by iDCs upon recognition of *A. fumigatus* conidia and hyphae (84, 97, 98).

##### Innate Lymphoid Cells and Innate-Like T Cells

Innate Lymphoid Cells (ILCs), γδ T cells, and mucosal associated invariant T cells (MAIT cells) have also been reported to have important roles in the innate immune response against *Aspergillus* spp. ILCs, and particularly type 3 ILCs (ILC3s), are commonly found in barrier epithelial surfaces, where they contribute to the maintenance of mucosal homeostasis, elimination of pathogens, regulation of the inflammatory response, as well as tissue remodeling (99). γδ T cells are innate-like T cells displaying features of both the innate and adaptive immune system. They are mainly involved in the immune surveillance and defense against pathogens in various peripheral tissues, including the lung (100). γδ T cells have been reported to produce IL-17A following challenge with *A. fumigatus* conidia (101). Moreover, mice lacking γδ T cells were more susceptible to *A. fumigatus* infection (102). Together with ILC3s, γδ T cells were shown to produce IL-22, which is critical for an efficient clearance of *A. fumigatus* (101, 103). Furthermore, deficiency in IL-22 production resulted in an impaired production of cytokines and chemokines and an impaired clearance of *A. fumigatus* in the murine lung (101, 103). MAIT cells are a subset of CD8+ unconventional T cells which are abundant in mucosal surfaces such as lung (100), and constitute up to 10% of the total T cells present in the peripheral blood (104). Upon activation, MAIT cells release proinflammatory cytokines such as IFNγ, TNFα, IL-17, and IL-22, and are able to kill pathogens through the production and release of cytotoxic compounds such as perforin and granzymes (100, 104). MAIT cells have been shown to be activated by T cell receptor (TCR)-dependent mechanisms through direct contact with antigen presenting cells such as dendritic cells in the case of bacteria and yeasts (105, 106), and TCR-independent mechanisms via cytokines in the case of viruses (107, 108). However, the activation of MAIT cells by filamentous fungal pathogens such *A. fumigatus* is not yet well-understood (104). Jahreis and colleagues showed that a fast TCR-dependent response was elicited by MAIT cells against several *Aspergillus* species, including *A. fumigatus*, *A. terreus*, and *A. flavus* (104). This response is characterized by the upregulation of activation markers, such as the CD69 antigen, and the release of cytotoxic compounds such as granzyme A and perforin (104). Finally, Natural Killer (NK) cells have also been shown to have a role in the innate immune defense against *A. fumigatus* (18).

##### Adaptive Immunity

Following a fungal encounter and the initial activation of the innate immune system, adaptive immunity is rapidly organized to clear the pathogen efficiently. Indeed, three different CD4+ T-helper cell lineages have been shown to play crucial roles in pulmonary aspergillosis: Th1, Th2, and Th17 (109–111). Th1 cells response is associated with protective immunity through the secretion of the pro-inflammatory cytokines TNFα and IFNγ, which promote antifungal activity of macrophages and neutrophils at the site of infection (110, 112, 113). Interestingly, Th1 cells have been shown to induce a fungus-specific Th1 immunity to an epitope of the *A fumigatus* cell wall glucanase Crf1. This antigen can be presented by DCs through three common major histocompatibility complex (MHC) class II alleles, which induces memory Th1 cells that are cross-reactive to *C. albicans* (114).

Alternatively, Th2 cells response is rather associated with non-protective immunity through the activation of M2 macrophages and decrease of Th1 response (110, 113). Th2 responses are predominant in patients suffering from ABPA, and are characterized by a decrease in IFNγ and an increase in IL-4 and IL-10 production, which in turn promotes humoral responses, through IgE production, and allergy (112, 113). Interestingly, fungal PAMPs have been shown to act as adjuvants enhancing T cell responses (115–117). This is notably the case of chitin, which is present in the fungal cell wall, in allergic disorders such as asthma (118–122). Dubey and colleagues showed that mice pre-treated with chitin before being challenged with *A. fumigatus* extract had high IgE levels (123).

The role of Th17 cell response is less clear. On the one hand, Zelante and colleagues showed that production of IL-17 and IL-23 by Th17 is not protective in a murine *A. fumigatus* infection model and inhibits Th1 cells development and antifungal activity of neutrophils *in vitro* (124). This was confirmed by blocking IL-17 and IL-23 production which showed increased clearance of *A. fumigatus*. This protective effect of IL-17 and IL-23 has been confirmed in an acute aspergillosis murine model, where mice were sensitized with *A. fumigatus* (125). The IL-17 and IL-23 -producing cells were identified by the authors as eosinophils (125). On the other hand, Jolink and collaborators showed that IL-17 provides a protective immunity by decreasing lung fungal burden in a murine infection model (126). Moreover, a cross-reactive Th17 response to *C. albicans* and *A. fumigatus* has also been described during acute ABPA in humans (127). This strong cross-reactive response is suggested to be rather induced by *C. albicans*-specific Th17 cells upon encounter with *A. fumigatus*, than by naïve T cells (127).

Lastly, regulatory T (Treg) cells have also been shown to have a protective effect in the immune response in aspergillosis. Treg cells have been shown to regulate the inflammatory response caused by a strong Th1 response in the early phase of *A. fumigatus* infection, as well as in the case of allergic reaction due to Th2 responses (113, 128, 129).

There is evidence of oligoclonal expansion of T cells upon exposure to *A. fumigatus*. For instance, stimulation with the allergen Asp f 1 induced an oligoclonal expansion of antigen-specific T cells directed to this antigen in ABPA and non-ABPA patients (130). Furthermore, the p41 epitope of the *A. fumigatus* extracellular cell wall glucanase Crf1 has also been shown to induce an antigen-specific oligoclonal T cell response in HSCT patients (114).

### Virulence Factors and Immune Evasion in *Aspergillus fumigatus*

*Aspergillus fumigatus* has evolved several virulence factors to escape innate immune responses. These virulence factors include the rodlet layer, DHN-melanin, ROS detoxifying enzymes, and toxins. Resting conidia are surrounded by a rodlet layer composed of hydrophobic RodA proteins (18, 80, 131, 132). This protein coat masks cell wall β-1,3-glucans, and thus prevents the detection of conidia by the innate immune response (69–71). Moreover, the rodlet layer was found to participate in the adherence of conidia to the pulmonary epithelium (131). DHN-melanin is the major melanin pigment that gives a gray-green color to *A. fumigatus* (133). It protects the integrity of the genome in conidia from ultraviolet light, as well as ROS (131, 134). It also masks fungal PAMPs, similar to the rodlet layer, and permits the fungus to evade phagocytosis by interfering with the acidification of the phagolysosome notably by interfering with intracellular Ca^2+^ signaling (135, 136). Moreover, *A. fumigatus* possesses a number of different enzymes for detoxifying ROS produced by host phagocytic cells such as macrophages. These enzymes include catalases, superoxide dismutase, glutathione transferases, fatty acid oxygenases, and efflux pumps, all of them either detoxifying H_2_O_2_ or superoxides, or expulsing ROS extracellularly (131, 134). Finally, *A. fumigatus* secretes several toxins considered as secondary metabolites, that further enable it to evade the immune response. These toxins are virulence factors crucial for *A. fumigatus* pathogenesis. For instance, gliotoxin has been shown to have several immunosuppressive effects, including inhibition of phagocytosis and neutrophil-derived ROS production, as well as proapoptotic activities (137, 138). Moreover, gliotoxin decreases ciliary movement and angiogenesis (131, 134, 139). All these immunosuppressive effects are thought to result from inhibition of the NF-κB signaling pathway (140), which as mentioned above is a key mediator of inflammatory responses (141).

### pH Modulation as a Strategy to Colonize the Host Tissues

**FIGURE 3:**
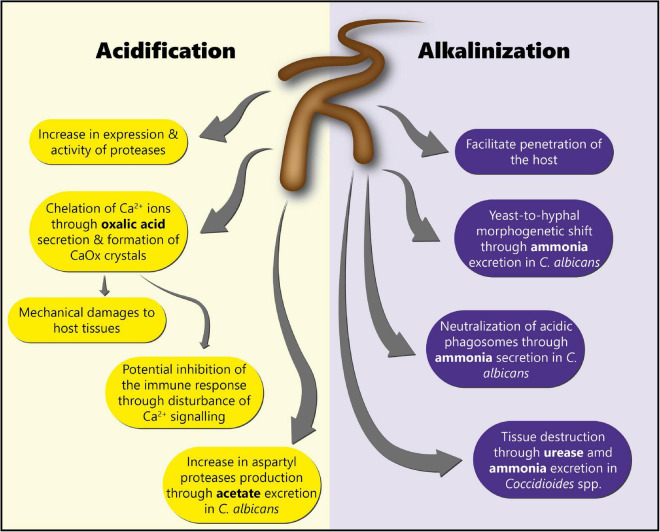
Roles of acidification and alkalinization mechanisms in fungal pathogenesis. This scheme summarizes the different roles of acidification and alkalinization mechanisms in fungal pathogenesis for the colonization of the host tissues.

Environmental pH is an extremely important factor influencing not only fungal growth and development, but also fungal physiology (142, 143). Indeed, pH modulation has been shown to affect fungal enzyme activity (144), and to be a crucial element controlling fungal pathogenicity. Fungal infections are often accompanied by a shift in pH in the surrounding host tissue (142), through the secretion of either acids or alkali (143). The specific roles of acidification and alkalinization in fungal pathogenesis discussed in this review are summarized in Figure 3.

Interestingly, fungi, including *Aspergillus* spp. *and Candida* spp. are known to produce low molecular weight organic acids (LMWOAs) such as oxalate, citrate, malate, formate, acetate, and succinate, which contribute to pH modulation (145, 146). Pathogenic fungi acidify their environment in order to enhance the activity of enzymes, as well as to damage the host tissues (143, 147). For instance, in the case of *C. albicans*, acidification through acetate excretion has been shown to allow the production of aspartyl proteases (143, 148–150), which are major virulence factors in this pathogen (151).

Oxalic acid is a known pathogenicity factor for the phytopathogenic fungi *Sclerotinia sclerotiorum* and *Botrytis cinerea* (152, 153). This acid is secreted in the host tissues and accumulates in the form of oxalate, leading to a pH decrease. Additionally, as oxalate is a strong chelator of divalent metallic cations, it can sequester calcium ions, with multiple possible structural and physiological consequences for the host (146). In the case of plant pathogens, the formation of calcium oxalate (CaOx) crystals in the middle lamella weakens the cell wall structure and facilitates infection. Moreover, oxalate can inhibit plant defenses and induce programmed cell death, which is also beneficial for necrotrophic pathogens (17, 146, 153).

The significance of a similar production of oxalic acid or other LMWOAs by *Aspergillus* spp. to infect the human lung is an active topic of research. Indeed, although many aspects of the ecology of *Aspergillus* spp. have been investigated in relation to their pathogenicity, one aspect that has been largely ignored is its ability to lower the pH of its environment. This may be necessary for their capacity to colonize or cause infection, via the secretion of oxalic acid or other low molecular weight organic acids. Several studies have reported the presence of CaOx crystals in the case of pulmonary aspergillosis (154–164), and the detection of CaOx crystals has been proposed as an easy tool for differential diagnosis (162). In most of the reported cases, oxalate deposition was associated with *A. niger* infection, but some reports also include infection caused by *A. flavus* or *A. fumigatus* (159). Oxalic acid and oxalate crystals are thought to cause host tissue damage (including in pulmonary blood vessels), as well as tissue injury via iron-dependent generation of free radicals (157, 163). A mechanical role of CaOx crystals was recently reported by Yi et al. (164) in a case of pulmonary angioinvasive aspergillosis in a Burkitt’s lymphoma patient with severe neutropenia, with pathophysiological examinations showing the presence of CaOx crystals around and within the walls of blood vessel. Aside from mechanical damage to the host tissues, the formation of CaOx could also have a dramatic effect on cell physiology. Indeed, calcium is an extremely important secondary messenger in many cell types, including those of the immune system (165). During immune stimulation, Ca^2+^ mobilization from extracellular medium or cellular compartments is essential to increase intracellular Ca^2+^ concentration (166), and thus Ca^2+^ chelation has been shown to inhibit the immune response *in vitro* (167, 168). All this suggests a potential role of oxalic acid also in the inhibition of the immune response. Despite these converging indications, a potential link between oxalic acid production and pathogenicity for *Aspergillus* spp. in animals has only been recently proposed (169, 170). Indeed, we demonstrated that oxalic acid production by *A. niger* led to a strong pH decrease, as well as calcium ion sequestration and precipitation in the form of CaOx crystals in differentiated 3D bronchial epithelial tissues. Moreover, we showed that the addition of the soil oxalotrophic bacterium *Cupriavidus oxalaticus* inhibited the growth of *A. niger*, and reverted pH values and free calcium concentrations back to physiological levels. Furthermore, CaOx crystals were no longer observed, suggesting the consumption of oxalic acid by *C. oxalaticus* (169, 170). However, the exact contribution of oxalic acid in the pathogenesis of *A. niger* still needs to be determined. Conversely, oxalic acid production by *Candida* spp. has never been confirmed experimentally, although these fungi are listed as oxalate-producers in the Human Metabolome Database (HMDB0002329).

Fungal pathogens are also known to manipulate the pH of their environment through alkalinization. This is notably the case of *C. albicans*. Alkalinization has been shown to facilitate the invasion of the host tissues, and the evasion of the immune system through neutralization of acidic macrophages phagosomes (150). Alkalinization of the host environment occurs through the excretion and accumulation of ammonia (NH_3_), which is then converted into ammonium ions (NH^4+^) by the urease (143, 150). Moreover, a lack of carbon is required for ammonia-mediated alkalinization to occur (142). Furthermore, *C. albicans* has been shown to auto-induce its switch to the hyphal growth form through the release of ammonia (171). *Coccidioides* spp. has been reported to excrete urease and ammonia to destroy the host tissue (172, 173). St Leger and colleagues have shown that *A. fumigatus* produced small amounts of ammonia in minimal medium, leading to a pH increase and allowing the production of active proteases (174). However, to the best of our knowledge, the role of environmental alkalinization in the pathogenesis of *A. fumigatus* has never been investigated.

## Lung Ecology

### Lung Homeostasis

Contrary to the gut, whose microbiota has been extensively studied, the lungs were considered sterile for a long time (175). However, the lung is now known to harbor a diverse microbiota composed of bacteria, fungi and viruses (176, 177). The bacterial composition in the lung has been studied in depth over the past years, although many studies have remained descriptive and in-depth mechanistic analyses are scarce. In the healthy lung, the microbiota is dominated by the genera *Prevotella*, *Streptococcus*, and *Veillonella* (178, 179). In patients with an acute or chronic respiratory disease the composition differs (180–183). Recent data provide clear evidence for the role of the airway microbiota (184), for instance, in the modulation of the host immune response or mucus production (185).

Whereas, the influence of bacterial community composition on lung homeostasis and disease has been a very active area of investigation, our knowledge on the involvement of fungi in these processes is still limited. Using fungal ribosomal RNA gene sequencing of BAL and sputum samples, it has been demonstrated that the mycobiota of the healthy lung mainly consists of environmental agents *Davidiellaceae*, *Cladosporium, Aspergillus, Eurotium, Penicillium*, and *Candida* (181). Epithelial cells of inner and outer body surfaces (e.g., intestine, skin, and lung) are the first physical barrier which interacts with commensals and are also important producers of antimicrobial peptides and immune mediators that regulate immune homeostasis and host defense (186, 187). Fungi colonize all barrier surfaces, and investigations into the influence of fungi on host immunity during homeostasis and disease are more advanced in the intestine and the skin. The influence of fungal seeding and colonization on immune maturation in the lung remains to be investigated, and we can learn from the findings in the intestine and skin and translate those to the lung in the future (188–190).

### Gut-Lung Axis

In-depth analysis of the microbiome has revealed profound differences in composition between various body compartments (191, 192). The importance of inter-organ communication and the role of the microbiome herein has been increasingly recognized (193). Respiratory diseases have not only been associated with microbial dysbiosis in the lung, but also in the gut (194). The gut microbiota composition has been shown to influence the immune responses of distant organs, including lungs, through the systemic dissemination of metabolites such as short chain fatty acids (SCFAs), or through the direct seeding of bacteria from the gut to the lungs by gastro-oesophageal reflux and microaspiration. This crosstalk between the gut and the lung compartment is called the gut-lung axis (195). The metabolites or bacteria can consequently have a stimulatory effect on the local immune cells (194–196), and ultimately impair or contribute to the development or progression of respiratory disease.

Recent studies in which fungal composition in the gut was altered using antifungal drugs, provide evidence for an immunoprotective role of the gut mycobiota (197, 198). For example, prolonged oral administration of antifungal drugs led to an exacerbation of allergic airway inflammation in an experimental mouse model. Antifungal treatment led to alterations in several fungal species, with an increase in *Aspergillus, Wallemia* and *Epicoccum* spp., and an observed decrease in *Candida* spp. The enhanced allergic airway inflammatory response was recapitulated when orally supplementing mice with a mixture of the three enriched fungi. This indicates that disruption of commensal fungi can influence both local and distal immune responses. Whether oral antifungal drugs also affect lung mycobiome composition is currently unexplored.

Disruption of bacterial communities using antibiotics can induce fungal dysbiosis and vice versa, suggesting an important role for inter-kingdom interactions (197). Indeed, the *bacterial* microbiome is likely to have an influence on the composition of the *fungal* microbiome, either directly through bacterial-fungal interactions or indirectly through its impact on host immunity, thus making the lung environment more permissive or restrictive to fungal growth (196). Interestingly, using a gnotobiotic approach, van Tilburg and colleagues demonstrated that the presence of gut bacteria, but not fungi, in early life could reduce allergic airway inflammation in a respiratory OVA sensitization and challenge model. These outcomes suggested that homeostatic control of allergic airway inflammation is dependent on bacterial presence, and that intestinal colonization with fungi can skew this inflammatory response (199).

Although a link between the lung bacterial microbiota composition and the disease outcome in patients with invasive pulmonary aspergillosis has been recently demonstrated (200), many knowledge gaps still exist regarding the link between the pulmonary microbiota and *Aspergillus* spp. infections. For instance, alteration of the air-blood barrier, and in particular enhanced access to the extracellular matrix, is a known risk factor for fungal infection (201), especially in the case of *Aspergillus* (202). Therefore, investigating the role of the lung microbiota and specific bacterial community compositions on the strengthening of the air-blood barrier is of clinical importance. The integration of principles from ecological theory will be key to elucidate the bacterial—fungal interactions of the gut and pulmonary microbiota and their human host. This will contribute to identifying microbial groups from within the airway microbiota, or their metabolites, for the development of therapeutic tools to control *Aspergillus* (179, 196).

## Current Therapeutic Strategies

The prompt diagnosis and treatment of invasive fungal pulmonary infections such as pulmonary aspergillosis is crucial to prevent associated complications and fatal outcomes. As the clinical presentations and radiological changes are non-specific, biopsy and histopathological analysis remains the gold standard for securing the diagnosis, but is frequently contraindicated in clinically marginal patients. Moreover, colonization is difficult to discriminate from a true invasive infection, and fungal blood cultures are insensitive (10, 203, 204). Therefore, serum and BAL biomarkers such as galactomannan and β-D-glucan, or PCR to detect fungi are being increasingly employed to establish the diagnosis (205).

Current available treatments for pulmonary aspergillosis are limited. They include the use of antifungal drugs—such as azoles, echinocandins, polyenes, and flucytosine, as primary treatment in the case of CPA or IPA, surgical resection in the case of patients suffering from aspergilloma and presenting associated complications such as severe hemoptysis, and corticosteroids in the case of ABPA, with or without the administration of antifungal drugs (206). The standard of care for fungal pulmonary infection is similar across different underlying lung diseases. Treatment would rather depend on the clinical manifestations: for example, no treatment in the case of colonization, corticosteroids and/or antifungal and/or newer biologics in case of allergic sensitisation/ABPA, and antifungal agents for invasive infection. The frequency by which these clinical manifestations are observed would depend on the underlying lung disease. For example, invasive infection is seen almost exclusively in immunocompromised patients, ABPA in patients with asthma or CF, whereas colonization can be observed in any lung disease associated with structural damage (COPD, CF, and non-CF bronchiectasis).

Resistance to antifungal drugs has increased dramatically in the last decades. While antibiotic resistance has been widely recognized in bacteria, antifungal resistance in opportunistic fungal pathogens has not yet received sufficient consideration. The reasons for the rise in antifungal resistance are multiple. Indeed, there is a limited arsenal of active antifungal compounds available on the market that are being used in both agriculture and human health, thus fostering the emergence and rapid spread of cross-resistance in human opportunistic fungal pathogens (2).

This is particularly well highlighted in the case of *A. fumigatus* resistance to azoles. Azoles are frontline antifungal compounds used in crop protection and in human and animal health (207, 208). They have a fungistatic effect on yeasts such as *Candida albicans*, while acting as fungicides against filamentous molds such as *A. fumigatus*. Their fungicidal effect against *A. fumigatus* is linked to defects in cell wall remodeling, resulting in loss of cell wall integrity and death (209). However, despite the fungicidal effect of triazoles in filamentous fungi, their application in agriculture at sub-inhibitory concentrations has led to the emergence and rapid spread of resistance among natural populations of *A. fumigatus* in soil (2, 210). This resistance to azoles may compromise the success of treatment in human patients suffering from *A. fumigatus* infections (211). Azole fungicides such as propiconazole, difenoconazole, or tebuconazole have a very similar structure when compared to those used in clinical practice, and their use is correlated with the increased emergence of clinical azole-resistant *A. fumigatus* strains (208). The emergence of this resistance led to the hypothesis that the extensive use of azole fungicides in agriculture selected for azole resistant *A. fumigatus* in the environment (212). This is supported by several studies reporting the presence of azole-resistant *A. fumigatus* strains in patients never treated with azoles (16, 213). This accounts for two thirds of patients suffering from azole-resistant aspergillosis. Resistance has been attributed to specific mutations in the tandem repeat (TR) of the *cyp51A* gene promoter region, which is involved in the biosynthesis of ergosterol in fungi (16, 214). Clinical azole-resistant *A. fumigatus* strains have also shown cross-resistance to azoles commonly used in agriculture (16). Thus, considering an integrated disease management approach through the One Health initiative that brings together scientists, medical doctors, veterinarians, and plant pathologists, is needed to reduce our reliance on chemical control alone and to stop the spread of resistance among opportunistic pathogens (2).

In case of pulmonary aspergillosis refractory to azoles, echinocandins, such as caspofungin or micafungin, or polyenes, such as amphotericin B, are used as second-line antifungal treatments (211, 215). Echinocandins inhibit the synthesis of the fungal cell wall component β-(1,3)-D-glucan by targeting the β-(1,3)-glucan synthase (216). Little is known on the resistance mechanism to echinocandins in *A. fumigatus*, due to its limited use in the treatment of Aspergillosis (216). Echinocandin resistance in *A. fumigatus* has been attributed to mutations in FKS genes encoding the β-(1,3)-glucan synthase (216). The same has been reported for *C. albicans* (217). Amphotericin B (AmB) has been shown to bind to ergosterol in the fungal cell membrane and form pores, thus disrupting the cell membrane integrity (218, 219). AmB has also been reported to induce endogenous production of ROS leading to oxidative stress and fungal death (219). Although resistance to AmB is rare, *A. terreus* has been shown to be intrinsically resistant (218). Moreover, AmB resistance has also been reported in *A. fumigatus* (220) and *A. flavus* (221). AmB resistance in *A. terreus* may be due to high endogenous production of catalase (218). Finally, flucytosine (5-FC) is a synthetic antifungal compound which, when it is taken up in fungal cells, is first converted into 5-fluorouracil (5-FU). 5-FU is then converted into metabolites which inhibits DNA and RNA synthesis (222). 5-FC is rarely administered as a monotherapy to treat fungal infections such as aspergillosis, but rather in combination with AmB (222, 223).

## Advances in Therapeutic Strategies and Future Perspectives

Current approaches used for the treatment of pulmonary aspergillosis focus on attacking the pathogen directly via the use of antifungal compounds. New antifungal drugs have been recently developed against *Aspergillus* spp. infection. Newly developed drugs, as well as drugs currently in development, are listed on the Aspergillosis website.^1^ Several of these have shown promising results and are being tested in phase 3 clinical trials (224). Olorofim (F901318, ClinicalTrials.gov Identifier: NCT05101187), from a new class of antifungal drugs called orotomides, is highly active against *Aspergillus* spp. It targets the dihydroorotate dehydrogenase (DHODH) which is involved in the pyrimidine biosynthesis pathway. Olorofim has been shown to inhibit conidia germination, as well as polarized hyphal growth (225). Biafungin, or Rezafungin (CD101, ClinicalTrials.gov Identifier: NCT04368559), is a novel echinocandin targeting the 1,3-β-D-glucan synthase. It shows high in vitro and *in vivo* activity against *Aspergillus* spp. (224, 226). Ibrexafungerp (SCY-078, MK-3118, ClinicalTrials.gov Identifier: NCT03059992) is a triterpenoid antifungal also inhibiting the biosynthesis of β-(1,3)-D-glucan (227). Ibrexafungerp has been shown to be effective against aspergillosis in an *in vivo* murine model (228). Finally, another interesting compound is ASP2397 (VL-2397), which is a novel natural antifungal compound currently in phase 2 clinical trial (ClinicalTrials.gov Identifier: NCT03327727). ASP2397 is a cyclic hexapeptide siderophore with a similar structure than ferrichrome, which has a high affinity for iron (224, 229). ASP2397 showed high antifungal activity against *A. fumigatus*, *A. terreus* and *A. flavus*. It was shown to inhibit conidia germination as well as hyphal growth. Moreover, ASP2397 has been reported to have a higher efficacy than posaconazole *in vivo* in an IPA mouse model (229). However, new therapeutic approaches are needed in order to slow down the pace of antifungal resistance emergence in fungal pathogens.

**FIGURE 4:**
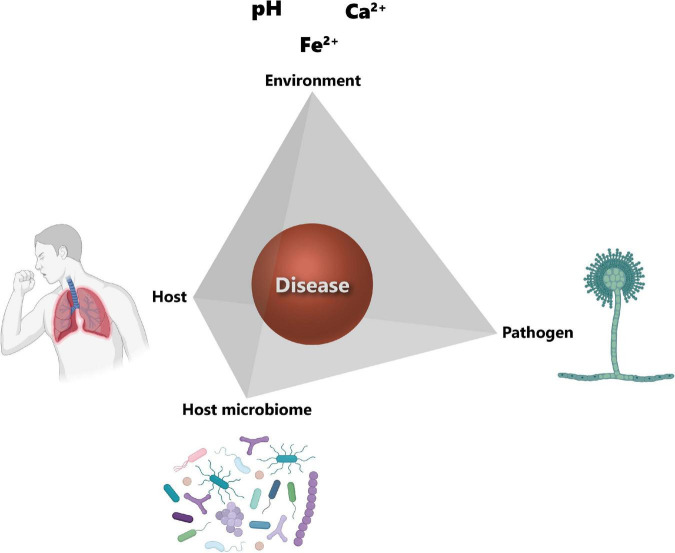
Disease pyramid. The onset of a disease depends on the interplay between the host, the pathogen, the environment, and the host microbiome. Host susceptibility mainly depends on genetic factors and immune status. The main factor for the successful colonization of the host tissues by the pathogen is its virulence. The environment corner refers to the host lung microenvironmental parameters and include among others pH, calcium (Ca^2+^) and iron (Fe^2+^) concentrations. Host microbiota community structure can also influence the establishment of the pathogen. Icons have been created using Biorender.com. Modified from Palmieri (170).

In order to cause disease, a pathogen needs a susceptible host, as well as suitable environmental conditions for its growth. These three factors, i.e., pathogen, host and environment, constitute the so-called disease triangle, which dictates the occurrence of a disease caused by a particular pathogen in a susceptible host in a particular environmental setting (152). The disease triangle concept has already been used in plant disease management for decades (230). However, one aspect that has been completely overlooked in this original disease triangle is the presence of the host microbiota and its role in disease development. A recent paper from Bernardo-Cravo et al. (231) highlighted the necessity to include the host microbiome as a fourth factor influencing the onset of a disease, as it plays a very important role in host immunity. Therefore, the disease triangle should become a disease pyramid (Figure 4) and one should consider all four factors, i.e., the host, the host microbiome, the environment, and the pathogen, when thinking about disease management.

We recently proposed to focus on the “environment” corner of the disease pyramid to control the growth of fungal pathogens such as *Aspergillus* spp. through a process we named “environmental interference” (169, 170). We demonstrated that the soil oxalotrophic bacterium *Cupriavidus oxalaticus* is able to control the growth of the fungal pathogen *A. niger*. This is achieved by degrading the oxalic acid produced by *A. niger*, preventing acidification of the local environment favorable to infection. Therefore, *C. oxalaticus* inhibits the growth of the pathogenic fungus *A. niger* by manipulating the pH and restoring it back to a more neutral, physiological value. This environmental interference principle could be potentially extended to other important environmental factors for fungal pathogenesis, such as iron. Indeed, iron is an essential nutrient that is generally limiting, and thus its acquisition is crucial for pathogen’s virulence (232). Ghio et al. (157) reported ferric iron (Fe^3+^) complexation at the surface of CaOx crystals associated with *A. niger* infection, resulting in lung tissue injury via the generation of oxidants. However, fungal, as well as bacterial pathogens, are well-known to acquire iron through the secretion of siderophores (233, 234). *A. fumigatus* is genetically equipped for efficient iron acquisition, encoding four different siderophores: fusarinine C (FsC), triacetylfusarinine C (TAFC), ferricrocin (FC), and hydroxyferricrocin (HFC) (235, 236). Experimental data showed that the initial phase of lung infection with *A. fumigatus* is accompanied by upregulation of iron acquisition genes (237). Loss in the ability to produce siderophores, and thus to acquire iron, has been shown to be detrimental for *A. fumigatus in vivo* (238). Moreover, interfering with the acquisition of iron by the use of chelators inhibited the growth of *A. fumigatus* in a murine cornea infection model (239). Therefore, developing a bacterial biocontrol strategy based on the interference of iron acquisition by *Aspergillus* would provide a further option to exploit the principle of interfering with the environment. This could be achieved either by direct competition between *Aspergillus* spp. and the biocontrol bacteria, where these latter would produce a siderophore with a higher affinity for iron than the fungal siderophore, allowing them to acquire iron better than the fungus, or by bacterial “cheating” through stealing the siderophores produced by *Aspergillus* spp. Indeed, non-siderophore producing bacteria are known to steal other species’ siderophores through the use of a matching receptor (240). On the other hand, host cells internalize iron through a global process called nutritional immunity, aimed at controlling infection (241, 242). However, unlike for the gut microbiota (243), the interplay between the regular members of the airway microbiota and nutritional immunity is still largely undetermined. Indeed, while essential metals, such as iron or zinc, have been found to be altered in several respiratory diseases, the exact causes and mechanistic consequences of this metal dysregulation on the immune system and respiratory microbiota still need to be further explored (242). Interestingly, gallium, a group IIIA metal, has been proposed as an antifungal agent. Gallium is used in several medical applications ranging from cancer to calcium disorders and bone metabolism (244). Moreover, gallium nitrate III [Ga(NO_3_)_3_] has been widely used as an antibacterial agent against bacterial pathogens such as *Klebsiella pneumoniae*, *Staphylococcus aureus* or *Pseudomonas aeruginosa* (244). Furthermore, gallium nitrate IV has been tested in a phase 2 clinical trial for intravenous administration as an anti-infective agent against *P. aeruginosa* infection in CF patients (ClinicalTrials.gov Identifier: NCT02354859). Gallium is known to disrupt the iron homeostasis in bacteria and cancer cells (244). Its antifungal inhibitory effect has recently been demonstrated by Bastos and colleagues against azole-resistant *A. fumigatus* and multidrug-resistant *Candida* spp. (244). Finally, it is worth investigating whether gallium could be used to treat bacterial-fungal polymicrobial infections, which are frequent in CF patients (245, 246).

The role of the microbiota as a keystone factor influencing the onset and development of a disease provides a powerful incentive for the use of microorganisms to prevent and treat illness. These microorganisms, also referred to as live biotherapeutic products (LBPs), are defined by the FDA as “*a biological product that contains live organisms, such as bacteria; is applicable to the prevention, treatment, or cure of a disease or condition of human beings; and is not a vaccine*” (247). They are not intended to reach the systemic circulation, but rather exert their action through interaction with resident members of the microbiota and/or by modulating complex host-microbiota interactions. This implies a multifactorial mode of action, which is in strong contrast to the reductionist approach traditionally used in medical research (247).

The large majority of LBPs correspond to single species products. There are examples in which single species LBPs appear to confer protection against an invading pathogen, a phenomenon known as colonization resistance [i.e., *Lactobacillus murinus* protection against *Streptococcus pneumoniae* lung colonization (248); *Clostridium scindens* providing colonization resistance against *Clostridium difficile* (249)]. However, the use of single-species LBPs likely fails to capture the complexity of the multifactorial and multi-species role of the microbiota in health homeostasis. A similar concept to colonization resistance is widely used in soil ecology, namely that of disease-suppressive and conducive soils (250). This concept links the composition of the soil microbiota with the natural protection against plant fungal infections. Mendes et al. (251) showed that a specific assemblage of rhizospheric bacteria lead to disease-suppression and confer protection against the root fungal pathogen *Rhizoctonia solani* in sugar beet seedlings. In the same way, a specific microbiota composition could lead to more pathogen-suppressive communities, or conversely, dysbiosis of the microbiota could lead to pathogen-permissive communities. Translating soil ecological concepts into human medicine could be of great benefit, as it would provide key insights into the complex interspecies interaction dynamics within the human microbiome and between itself and the host (252, 253). Such lung microbiota-based biocontrol strategy could be applied against aspergillosis, as lung microbiota has been suggested to prevent the establishment of *Aspergillus* spp. in the lungs (196).

Additionally, LBPs have been shown to restore the epithelial barrier and modulate the immune response. These effects have been mostly studied in the case of administration of probiotics in the gastrointestinal tract (254). However, their potential beneficial effect on the airway epithelial barrier restoration, as well as in the immune homeostasis have been suggested by Martens et al. (254). This is notably the case of the bacterial strains *Lactobacillus plantarum* MB452, *Lactobacillus rhamnosus* GG and *Streptococcus thermophiles* ATCC 19258, among others (254). Recently, the use of nasal probiotic strains such as *Lacticaseibacillus casei* AMBR2 and *Lactococcus lactis* W136 has shown promising results. Indeed, *Lacticaseibacillus casei* AMBR2 showed a beneficial effect on the epithelial barrier function and modulation of the immune response in *in vitro* Calu-3 differentiated tissue co-cultured with donor-derived nasal microbiota and macrophage-like cells (255, 256). Cho and colleagues reported that *Lactococcus lactis* W136 suppressed the growth of patient-derived strains of the bacterial pathogen *Pseudomonas aeruginosa* in *in vitro* co-cultures (257).

Although large efforts have been made to enhance our understanding of respiratory fungal infections, the treatment of fungal pathogens poses a series of unique challenges. Fungal infections are difficult to diagnose in a timely manner, antifungal drugs and treatments are limited, and given the emergence of resistance to antifungal drugs there is a reduced effectiveness of these treatments. Moreover, severe infections often occur in subjects with significant comorbidities, including chronic respiratory diseases and a state of immunosuppression. It is thus imperative to develop novel treatment strategies for fungal infections. In order to do so, we have identified several *key unanswered questions* (Box 1). In view of the variability of fungal infection outcomes, and in particular those with *Aspergillus* spp., there is an urgent need to understand the conditions that make the respiratory tract permissive to conidial germination in susceptible individuals, and in particular, to determine whether the composition of the airway microbiota plays a role in this regard.

**Box 1. Key unanswered questions.**

° What comprises a healthy lung mycobiome?
° What is the role of the mycobiome component of the respiratory microbiota in the colonization resistance to fungal pathogens?
° What is the role of commensal fungi in microbiota community stability?
° What is the involvement of commensal fungi in the maintenance of lung barrier homeostasis/function?
° How do commensal fungi contribute to pulmonary immune maturation in early life and what are the consequences of early life fungal dysbiosis for respiratory disease development?
° Which mechanisms underlie the bacterial -induced predisposition to invasive pulmonary aspergillosis and other respiratory fungal infections?
° How does nutrient availability contribute to lung microbial community composition and consequently influence fungal infection?

Future research in this field should focus on moving toward the complete characterization of the lung microbial ecosystem by shotgun metagenomic sequencing and gene expression analysis (258). Moreover, technical advances are required to enhance our ability to culture fungi from respiratory tract samples in the setting of prophylactic anti-fungal treatment, and in particular move toward building a collection of lung commensal fungal strains which can be implemented in mechanistic studies.

## Author Contributions

FP and NU conceptualized and drafted the manuscript. AK, EB, PJ, and CG provided valuable discussion and input. FP, AK, EB, PJ, CG, and NU revised the manuscript. All authors approved the final version of the manuscript.

## Conflict of Interest

The authors declare that the research was conducted in the absence of any commercial or financial relationships that could be construed as a potential conflict of interest.

## Publisher’s Note

All claims expressed in this article are solely those of the authors and do not necessarily represent those of their affiliated organizations, or those of the publisher, the editors and the reviewers. Any product that may be evaluated in this article, or claim that may be made by its manufacturer, is not guaranteed or endorsed by the publisher.

## Footnotes

1
https://www.aspergillus.org.uk/
